# The Shark Assemblage at French Frigate Shoals Atoll, Hawai‘i: Species Composition, Abundance and Habitat Use

**DOI:** 10.1371/journal.pone.0016962

**Published:** 2011-02-10

**Authors:** Jonathan J. Dale, Austin M. Stankus, Michael S. Burns, Carl G. Meyer

**Affiliations:** 1 Hawaii Institute of Marine Biology, University of Hawaii at Manoa, Coconut Island, Kaneohe, Hawaii, United States of America; 2 Department of Zoology, University of Hawaii at Manoa, Honolulu, Hawaii, United States of America; University of Glamorgan, United Kingdom

## Abstract

Empirical data on the abundance and habitat preferences of coral reef top predators are needed to evaluate their ecological impacts and guide management decisions. We used longline surveys to quantify the shark assemblage at French Frigate Shoals (FFS) atoll from May to August 2009. Fishing effort consisted of 189 longline sets totaling 6,862 hook hours of soak time. A total of 221 sharks from 7 species were captured, among which Galapagos (*Carcharhinus galapagensis*, 36.2%), gray reef (*Carcharhinus amblyrhynchos*, 25.8%) and tiger (*Galeocerdo cuvier*, 20.4%) sharks were numerically dominant. A lack of blacktip reef sharks (*Carcharhinus melanopterus*) distinguished the FFS shark assemblage from those at many other atolls in the Indo-Pacific. Compared to prior underwater visual survey estimates, longline methods more accurately represented species abundance and composition for the majority of shark species. Sharks were significantly less abundant in the shallow lagoon than adjacent habitats. Recaptures of Galapagos sharks provided the first empirical estimate of population size for any Galapagos shark population. The overall recapture rate was 5.4%. Multiple closed population models were evaluated, with Chao M_h_ ranking best in model performance and yielding a population estimate of 668 sharks with 95% confidence intervals ranging from 289–1720. Low shark abundance in the shallow lagoon habitats suggests removal of a small number of sharks from the immediate vicinity of lagoonal islets may reduce short-term predation on endangered monk seal (*Monachus schauinslandi*) *pups*, but considerable fishing effort would be required to catch even a small number of sharks. Additional data on long-term movements and habitat use of sharks at FFS are required to better assess the likely ecological impacts of shark culling.

## Introduction

Sharks were historically one of the most abundant top predators in coral reef ecosystems but their numbers have declined in recent decades due to overfishing and habitat degradation [1], [2]. With the continuing deterioration of coral reefs worldwide, the few remaining pristine coral reef ecosystems provide a valuable opportunity to obtain baseline information on shark ecology useful for evaluating human impacts on exploited coral reef systems. The Papahānaumokuākea Marine National Monument (PMNM) consists of a series of rocky pinnacles, atolls, reefs and submerged banks extending 1,930 km northwest of the Main Hawaiian Islands (MHI), and represents one of the few remaining near-pristine coral reef ecosystems. Sites within the monument are uninhabited, off limits to fishing and characterized by high predator (mainly sharks and jacks) abundance [3]. These ecosystems are minimally affected by human impacts and are thought to represent the natural state of coral reef ecosystem structure [1], [3], [4]. In comparison, diver visual surveys suggest coral reef ecosystems of the populated MHI are dominated by herbivorous fishes and lower trophic level carnivores, with apex predators representing a minor component of the total fish biomass [3], [5]–[7]. The PMNM is therefore an ideal location to study the assemblage structure and habitat use of coral reef associated sharks in a minimally impacted environment. Empirical data of this type are needed to better understand the ecology of Hawaiian atolls, and to guide management decisions at these remote locations.

Detailed information on shark assemblages at remote atolls is scarce because of the logistical challenges associated with accessing these locations. Previous research quantifying shark abundance and species composition in the PMNM has consisted of short-term sampling periods (a few days at each island or atoll) over the course of one to several years [3], [8], [9]. Methods used by these studies have been aimed at maximizing spatial coverage of a broad range of genera (i.e. sharks and teleosts), yet each method contains inherent sampling bias. The primary method of estimating abundance has been underwater visual surveys (UVS; belt transects and towed diver surveys). The advantage of visual methods is the ability to survey large areas in a relatively short amount of time, yet there are inherent limitations associated with these techniques. Limitations include interspecific variability in behavioral responses to divers (some species flee, others approach divers), limited visual field of divers, inaccurate species identification of morphometrically similar species, and a limited survey depth range (<30 m). These limitations can create bias in diversity, abundance and size estimates [10]–[12] and can be especially problematic for large mobile species such as sharks [13]. In order to examine the validity of underwater visual techniques for estimating shark abundance, comparison with alternative sampling methods is required.

We conducted 3 months of intensive longline sampling to quantify the abundance, type and habitat use patterns of sharks at French Frigate Shoals (FFS) atoll. Our specific objectives were to (1) determine the diversity and relative abundance of shark species; (2) determine how abundance varies with habitat; (3) examine the size and sex structure of dominant species; (4) compare results between our longline and previous underwater visual surveys in estimating abundance, species composition and habitat use of sharks, and (5) calculate a mark-recapture based estimate of Galapagos shark (*Carcharhinus galapagensis*) population size at FFS.

## Methods

### Study site

**Figure 1:**
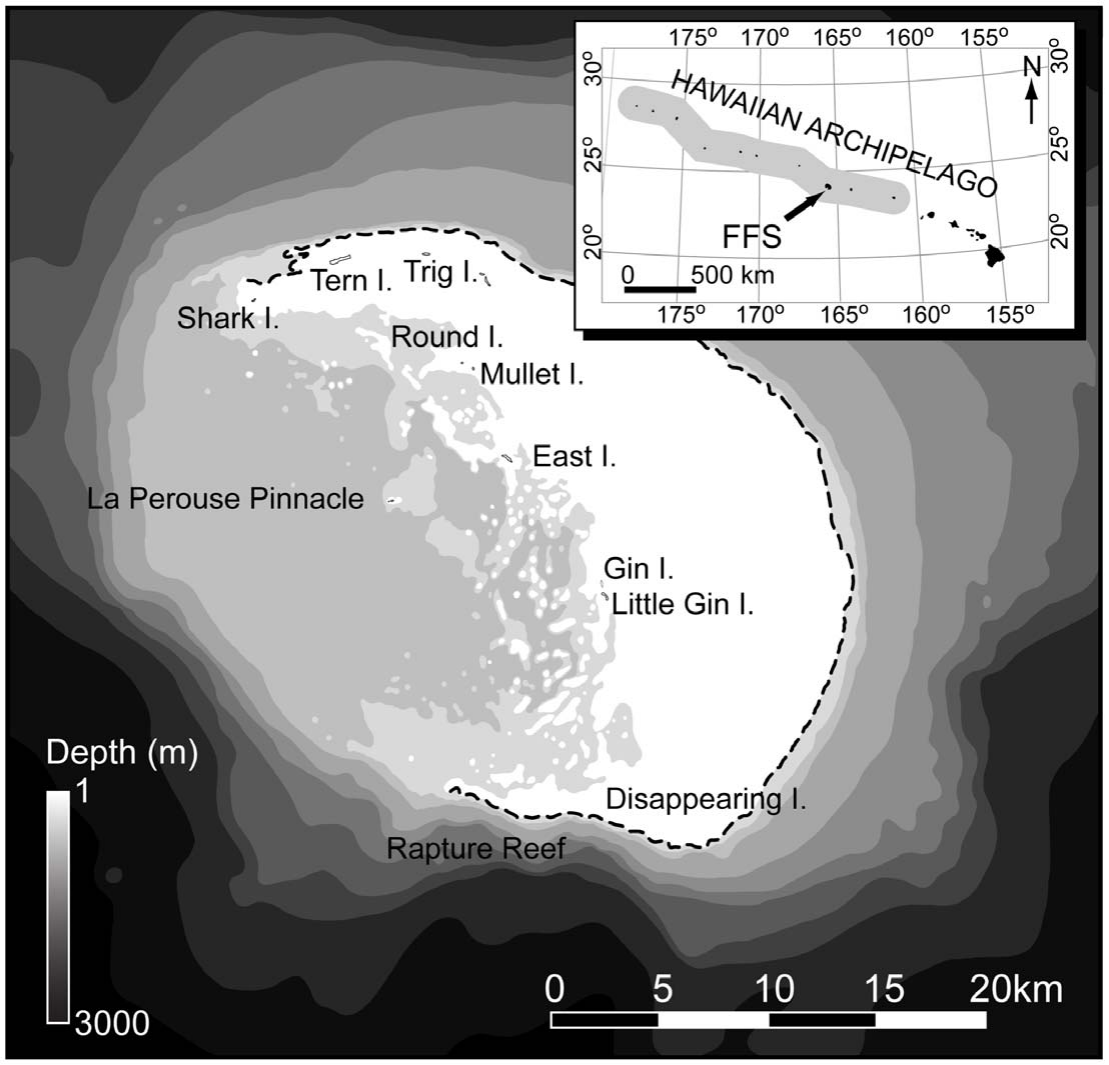
Map of Study Area. French Frigate Shoals atoll within the Papahānaumokuākea Marine National Monument (shaded area inset).

French Frigate Shoals (N23° 45′ W166° 10′) is located in the middle of the Hawaiian archipelago (Fig. 1). The atoll consists of a 34 km long oval platform bounded on the east side by a 50 km long crescent-shaped barrier reef (Fig. 1). Habitat outside the barrier reef consists of classical spur and groove formations running from the reef crest down to depths of 20–30 m. The western half of the atoll is open to the ocean and shelves gradually from depths of 20 to 100 m over a distance of 18 km, before descending more steeply to >1000 m depth. The eastern half of the atoll consists of a shallow (<1 to 10 m deep) lagoon enclosed between the outer barrier and an inner crescent shaped reef, and is 12 km wide at its midpoint. Lagoonal habitats include reticulate and patch reefs, submerged sand and coral rubble, and small sandy islets. Total coral reef area of the shoals is >940 km^2^ and total land area of the sandy islets is 0.25 km^2^.

### Fishing and Tagging

Capture and tagging of sharks during this study was approved and performed according to the Institutional Animal Care and Use Committee of the University of Hawai‘i Permit # 05-053-4 and Papahānaumokuākea Marine National Monument Research Permit # PMNM-2009-037. Shark surveys were conducted between May and August 2009, overlapping seasonally with previous UVS in the PMNM [3], [9]. Fishing gear consisted of 10 hook polypropylene longlines (440 m length) [14], set on the bottom in depths of 3 to 60 m. Branch lines consisted of 3 m of polypropylene rope connected via a large swivel to 1.5 m of braided stainless-steel cable leader terminating in a 20/0 gauge Mustad^TM^ circle hook. Branch lines were connected to the mainline with a snap clip at intervals of 40 m and baited with large tuna heads and fish scraps. Fishing gear was standardized for all sets during the duration of the study. A few sets towards the end of the study consisted of smaller fish and squid baits. These sets were not included in calculation of relative abundances, but sharks captured during these sets were included in overall assessments of species composition. Longline sets were conducted during daylight hours. Sets began approximately one half hour after sunrise and were either reset in the afternoon or checked every few hours throughout the day. Fishing location was highly dependent on daily weather conditions, preventing complete randomization of stations, but stations within accessible locations were randomly selected. Captured sharks were brought alongside a 6 m skiff, where they were tail-roped and inverted to initiate tonic immobility. Sharks remained docile in this position while they were measured, sexed and tagged with an external identification tag (Hallprint^TM^ stainless steel dart ‘wire through’ tags). Clasper length and degree of calcification was measured as an indicator of sexual maturity in males. The hook was then removed and the shark released. The entire handling process for each shark took less than 10 minutes.

### Data analyses

The statistical measure of abundance was catch per unit effort (CPUE), defined as the number of sharks caught per 10 hook hours. Fishing effort was stratified into 3 sub habitats: (1) shallow lagoon; 0–10 m depth with focus on habitat in proximity to islands, (2) deep lagoon; 11–30 m depth up to several km away from islands but still within the lagoon, and (3) outer reef; 30–60 m depth. CPUE data were non-normally distributed thus Kruskal-Wallis nonparametric tests were used to evaluate the effects of set time (morning vs. afternoon) and habitat on CPUE. Habitat effects were evaluated for all sharks combined and individually for common species. Significant habitat effects were evaluated *post hoc* with Dunn's pairwise comparisons. Sex ratios were tested for a departure from equality with χ^2^ tests for goodness of fit. Analysis of Variance (ANOVA) was used to test for significant differences in size structure of individual species between habitats.

Recaptures of marked Galapagos sharks allowed for estimates of population size. Estimates were made with the program CAPTURE [15]–[17] run as a subroutine within program MARK [18]. Several closed population models were used to generate abundance estimates [19]. The first model (M_o_) assumed constant probability of capture. Three additional models which relax the assumption of equal probability of capture were also considered: (1) variability in individual capture probability using the Chao and Jackknife M_h_ estimators [20], [21], (2) time-varying capture probability using the Chao and Darroch M_t_ estimators [17], [22], [23], and (3) behavioral response to capture using the Zippin M_b_ estimator [24]. A model selection routine within the CAPTURE program was used to rank each of the potential models. Model selection was based on seven goodness of fit tests used to evaluate the assumptions associated with each model. Ranks were derived from a multivariate discriminant function analysis based on results from the goodness of fit tests [19].

## Results

### Relative abundance and habitat use

**Figure 2:**
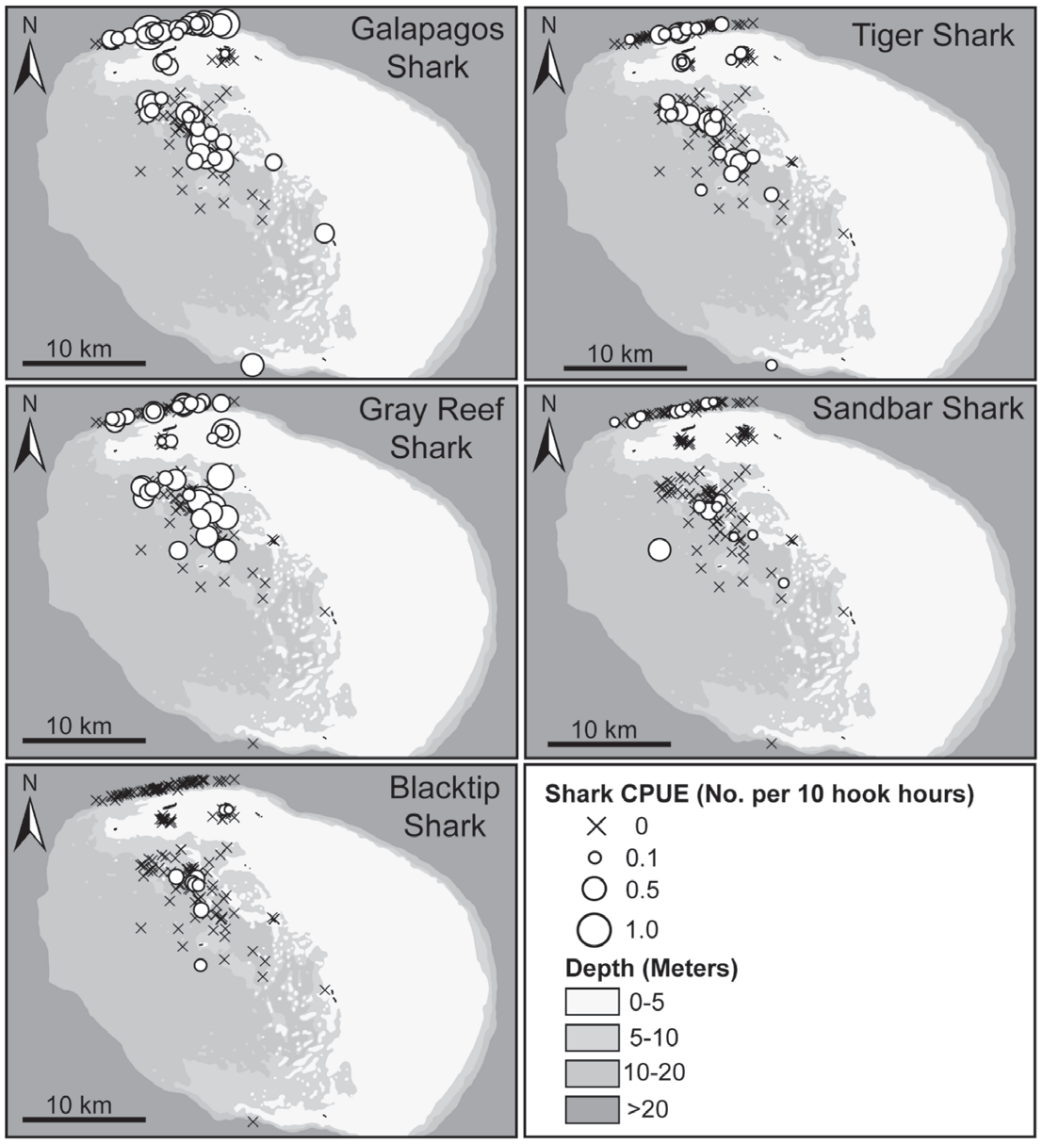
Spatial distribution of longline Catch Per Unit Effort (CPUE). In all panels, scaled circles indicate CPUE >0, crosses indicate CPUE  = 0. CPUE  =  sharks per 10 hook hours.

Between May and August 2009, longlines were set on 189 occasions, totaling 6,862 hook hours of soak time. A total of 221 sharks from 7 species were captured (Table 1). Although fishing was conducted throughout the entire atoll, the majority of effort was restricted to the northern half due to logistical limitations of operating from the Tern Island field station (Figs. 1, 2). The number of sets varied by month for both the deep lagoon and outside the barrier reef, due to weather restrictions on access to fishing locations. There was no significant difference in the number of monthly sets in the more protected and accessible shallow lagoon sites. The average duration of longline sets was longer in the shallow lagoon (6.2±2.7 h) than deep lagoon (4.0±0.8 h) and outer reef (4.0±1.2 h) sites. However, longer shallow lagoon sets were checked after approximately 4 h. There were no significant differences in CPUE between morning sets and afternoon sets for individual shark species or all species combined (P>0.05). Galapagos (36.2%), gray reef (*Carcharhinus amblyrhynchos*, 25.8%) and tiger (*Galeocerdo cuvier*, 20.4%) sharks were the numerically dominant species accounting for 82% of all sharks captured (Table 1). Sandbar (*Carcharhinus plumbeus*) and blacktip (*Carcharhinus limbatus*) sharks were captured less frequently, representing 10.4% and 4.1% of total shark catch respectively (Table 1). Species rarely encountered included scalloped hammerhead (*Sphyrna lewini*, N = 2, <1%) and whitetip reef sharks (*Triaenodon obesus*, N = 5, 2.3%) (Table 1).

**Table 1:** Summary of longline fishing data.

Habitat	Hook Hours	Galapagos	Tiger	Gray Reef	Sandbar	Blacktip	Other*	All Sharks
**OB** *	2533	45 (46.9)	16 (16.7)	18 (18.8)	15 (15.6)	0	2 (2.1)	96
**DL** *	2470	27 (28.1)	22 (22.9)	31 (32.3)	8 (8.3)	7 (7.3)	1 (1.0)	96
**SL** *	1859	8 (27.6)	7 (24.1)	8 (27.6)	0	2 (6.9)	4 (13.8)	29
**Total**	6862	80 (36.2)	45 (20.4)	57 (25.8)	23 (10.4)	9 (4.1)	7 (3.2)	221

Shark numbers indicate the sum of all sharks caught combined and by habitat. Values in parentheses are row percentages of all sharks.

*Other species include scalloped hammerhead and whitetip reef sharks,

*OB: Outside Barrier Reef,

*DL: Deep Lagoon,

*SL: Shallow Lagoon.

**Figure 3:**
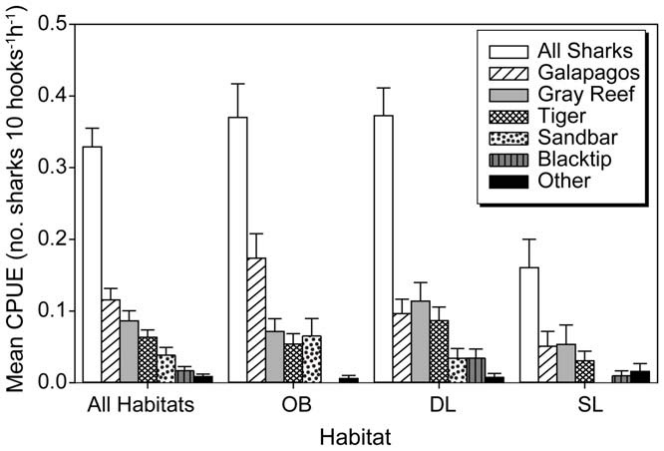
Mean Catch Per Unit Effort (CPUE) for shark species within habitats and overall. Shaded bars represent CPUE within habitats, unshaded bars represent overall CPUE. OB: Outside barrier reef; DL: Deep lagoon; SL: Shallow lagoon; CPUE  =  sharks per 10 hook hours.

Overall CPUE (all sharks combined) varied significantly by habitat (Kruskal-Wallis, H = 11.0, P = 0.004) (Fig. 3). Within habitats, CPUE in shallow lagoon areas (mean CPUE  = 0.15±0.25) was significantly lower (P<0.01) than other locations, but there were no significant differences between deep lagoon areas (mean CPUE  = 0.39±0.35) and outside the barrier reef (mean CPUE  = 0.37±0.39). Galapagos, tiger and gray reef sharks were captured in all three habitats, whereas sandbar sharks were not captured in the shallow lagoon and blacktip sharks were not captured outside the barrier reef (Fig. 2, 3). Both of the scalloped hammerhead sharks were captured outside the barrier reef and 4 of 5 whitetip reef sharks were captured in the shallow lagoon. Of the three numerically dominant species, only catch rates of Galapagos sharks varied significantly among habitats (H = 7.13, P = 0.028). Catch per unit effort outside the barrier reef (mean CPUE  = 0.17±0.28) was significantly higher (P = 0.012) than shallow lagoon areas (mean CPUE  = 0.04±0.12), but there were no significant differences between deep (mean CPUE  = 0.10±0.19) and shallow lagoon, or between deep lagoon and outside the barrier reef.

### Size, sex ratios and maturity

**Figure 4:**
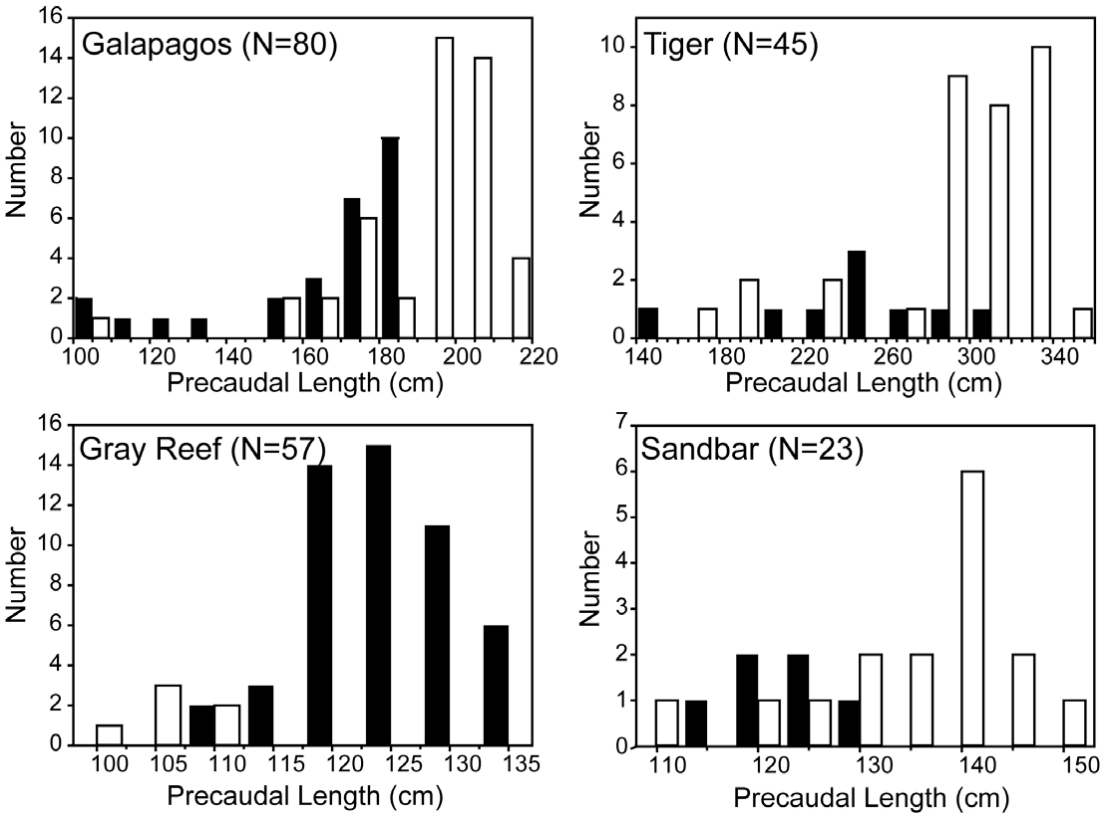
Size frequency of sharks captured at French Frigate Shoals. In all graphs, Black bars represent males, white bars represent females.

The size distribution of all species captured was skewed towards larger animals (Fig. 4). This is likely an effect of gear selectivity, as large hooks and baits were used specifically to minimize teleost bycatch. However, a wide size range of sharks were captured, including juveniles of the dominant species (Fig. 4). The majority of species showed no significant difference in size between habitats, but sandbar sharks were significantly larger in the deep lagoon (mean PCL  = 137.9±8.1 cm) compared to outside the barrier reef (mean PCL  = 128.4±10.6 cm) (ANOVA, F  = 4.78, df = 1, 20, P = 0.041). Sex ratios were significantly skewed towards females for Galapagos (1.7∶1), tiger (3.8∶1) and sandbar (2.7∶1) sharks, and towards males for gray reef (8.5∶1) sharks (Table 2). No bias in the observed sex ratio was evident for blacktip (1.3∶1) sharks (Table 2).

**Table 2:** Sex ratios for the five most common species of sharks at French Frigate Shoals atoll.

Species	Male	Female	Ratio	χ^2^	P*
**Galapagos**	27	46	1.7∶1	4.9	0.026
**Tiger**	9	34	3.8∶1	14.5	<0.001
**Gray Reef**	51	6	8.5∶1	35.5	<0.001
**Sandbar**	6	16	2.7∶1	4.5	0.030
**Blacktip**	5	4	1.3∶1	0.1	0.369

*P values in bold for χ^2^ tests are statistically significant.

Four (5.4%) of the 73 tagged Galapagos sharks were recaptured, yielding closed-system population size estimates ranging from 104 to 695 sharks (Table 3). The individual variability in capture probability model (M_h_) was ranked highest by the model selection procedure (Table 3). Two different estimators of individual variability (M_h_) were modeled, Jackknife M_h_ and Chao M_h_. The population estimate for the Jackknife M_h_ estimator was lower than the Chao M_h_ estimator (371 vs. 668 respectively) and had a narrower confidence interval (289–484 vs. 289–1720 respectively) (Table 3). The constant capture probability model (M_o_) was also ranked highly with a population estimate (695) and confidence interval (314–2180) similar to the Chao M_h_ model (Table 3). The behavioral response model (M_b_) had less support with the lowest population estimate (104) and narrowest confidence interval (83–172) (Table 3). No support was found for the time-varying capture probability model (M_t_).

**Table 3:** Population estimates for Galapagos sharks at French Frigate Shoals atoll from closed population models.

Model	Rank	N	S.E.	95% CI
Chao M_h_	1.00	668	331.2	289–1720
Jackknife M_h_	1.00	371	49.3	289–484
Null M_o_	0.97	695	332.7	314–2180
Zippin M_b_	0.77	104	20.2	83–172
Darrock M_t_	0.00	676	320.3	301–1674
Chao M_t_	0.00	528	232	251–1240

## Discussion

**Figure 5:**
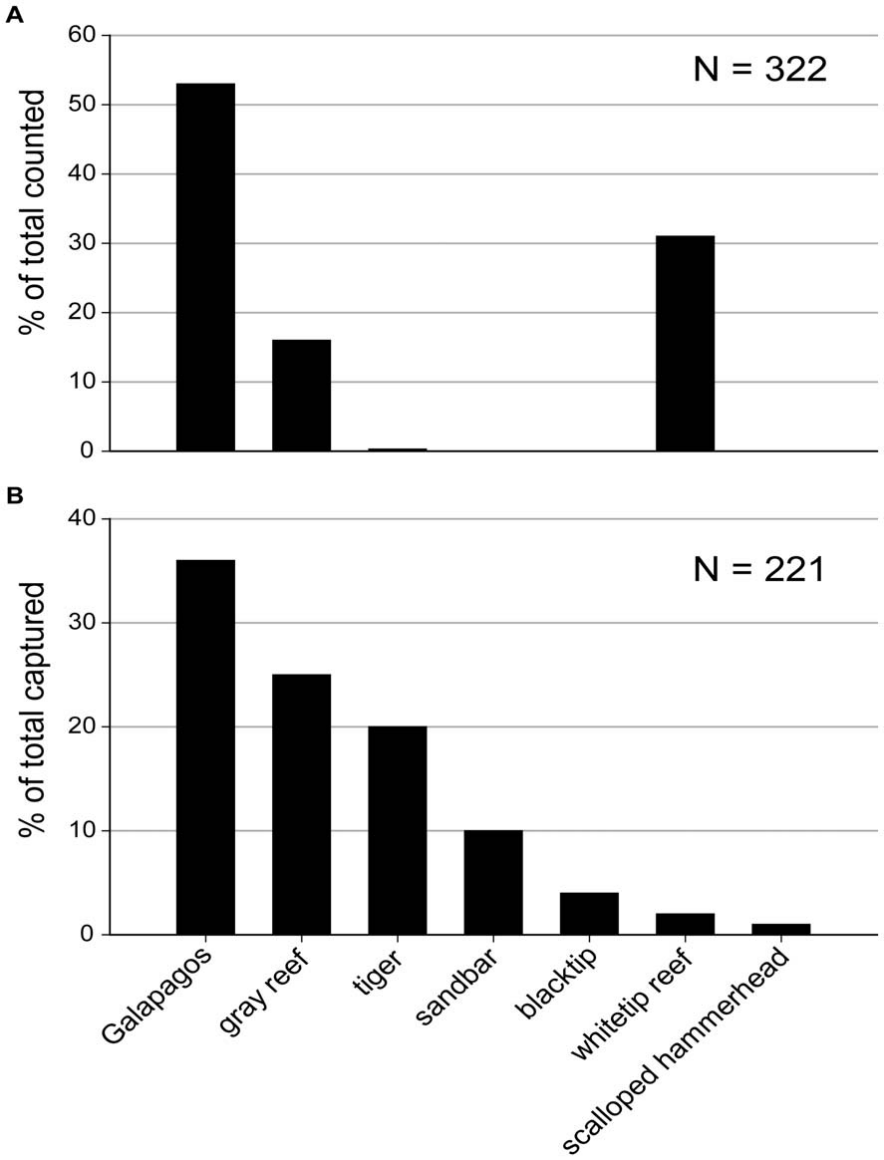
Comparison of relative abundances of sharks determined from underwater visual surveys and longline fishing surveys. Species composition of the shark assemblage obtained by (A) towboarding (underwater visual census, adapted from [9]) throughout the Papahānaumokuākea Marine National Monument and (B) shark longline fishing surveys at French Frigate Shoals atoll.

Comparison of our results with those of previous UVS studies conducted during summer within the PMNM (e.g. [3], [9]) suggests both methods introduce sampling bias but longline surveys provide a far more comprehensive picture of shark assemblage composition. Thus although both UVS and longline sampling found Galapagos sharks to be the most abundant shark species at FFS, UVS drastically underestimates the abundance of tiger, sandbar and blacktip sharks, whereas only whitetip reef sharks were underrepresented in longline catches (Fig. 5). Only a single tiger shark was recorded by UVS methods throughout the NWHI [9], whereas we captured 45 tiger sharks (20% of all sharks caught) at FFS and previous longline surveys also found high abundances of tiger sharks at this location [25]. UVS methods recorded no sandbar sharks anywhere in the NWHI, yet this species accounted for 10% of all sharks captured on our longlines at FFS (Fig. 5). Whitetip reef sharks were the second most abundant shark documented with UVS (Fig. 5, [3], [9]) yet were rarely caught by our longlines. Cumulatively, our results suggest UVS may not be a reliable method for estimating abundance of large sharks, and UVS studies which find low shark abundances may be fundamentally flawed.

Differences in estimates from UVS and longline methods may be behaviorally mediated, with some species (e.g. tiger sharks) actively avoiding divers, while sedentary daytime behavior of others (e.g. whitetip reef sharks, [26]) makes them highly susceptible to visual survey methods and less vulnerable to daytime longline fishing. Low whitetip reef shark abundances in our longline survey may also be due to gear selectivity (we used large hooks and baits not well-suited to catching whitetip reef sharks). Four of 5 whitetip reef sharks captured were caught on smaller baits towards the end of the study, suggesting a combination of gear types (i.e. both large and small hooks and baits) should be used in order to obtain a more complete representation of shark composition and abundance in coral reef ecosystems.

The diversity of shark species captured at FFS was broadly similar to that of other isolated atolls in the Indo-Pacific where a few species numerically dominate the shark assemblage [4], [27]–[32]. A lack of blacktip reef sharks (*Carcharhinus melanopterus*) distinguishes the shark assemblage at FFS from those at many other Indo-Pacific atolls where this species is abundant [27], [33], [34]. Galapagos, gray reef and tiger sharks were the numerically dominant species in our catches at FFS, and previous longline surveys conducted in Hawai‘i between 1978 and 1980 captured a similar species assemblage and found relatively high abundances of Galapagos and gray reef sharks in the NHWI compared to the MHI [25], [35]. There are currently no directed fisheries targeting (or significant bycatch of) coastal sharks in Hawai‘i, hence a lack of shark fisheries in the uninhabited NWHI does not adequately explain the greater abundance of certain shark species in this area. A feasible alternate explanation is differences in shark abundance between these two regions primarily reflect variations in both habitat preferences and prey abundances. For example, both grey reef and sandbar sharks are found throughout the Hawaiian archipelago but have opposite patterns of regional abundance, with grey reef sharks most abundant at NHWI atolls and sandbars most abundant around MHI high islands [25]. Additionally, fish biomass in the NWHI is up to 260% greater than the MHI [3], potentially limiting shark density in the MHI.

Our habitat-stratified sampling design provided higher resolution information on shark habitat preferences at FFS than previous longline surveys. For example, our CPUE data support results of previous visual census studies [9] indicating sharks are generally less abundant in the shallow lagoon than other habits at FFS. Low habitat diversity, absence of shallow lagoon specialists (such as blacktip reef sharks) and competition with teleost predators for prey resources may all be factors contributing to this pattern. NWHI atolls lack mangrove and seagrass habitats typically found at atolls with high abundances of blacktip reef sharks in shallow lagoons [4], [27], [33], [34]. Prey resources in shallow lagoon habitats at FFS may be insufficient to support high numbers of predatory teleosts (e.g. giant trevally, *Caranx ignobilis*) as well as sharks.

Although significant differences in CPUE among habitats were only detected for Galapagos sharks, a general preference for deeper atoll habitats was suggested for all shark species captured at FFS. Galapagos shark CPUE was significantly higher in the deep lagoon and outer reef habitats compared to shallow lagoon habitats, with the highest catch rates occurring outside the barrier reef. Catch rates of gray reef and tiger sharks were highly variable, but CPUE was highest outside the barrier reef and in the deep lagoon for both of these species. Sandbar sharks were only encountered in deep lagoon and outer reef habitats, and blacktip shark CPUE was highest in the deep lagoon. These patterns of habitat use are congruent with results of previous studies [36]–[39].

Sexual segregation is common in shark populations [40], and in most cases our observations at FFS are consistent with those of previous studies. The exception was gray reef sharks for which previous studies have documented female skewed sex ratios [41], [42]. Taylor [41] observed aggregations of female gray reef sharks in shallow waters at Laysan Island (NWHI), contrasting with our finding that male gray reef sharks predominated in shallow waters at FFS. These apparently conflicting results emphasize that sex segregation in sharks is a dynamic phenomenon, the timing and location of which may be determined by a suite of environmental and behavioral factors [42], [43].

The recapture of several tagged individuals enabled us to generate the first empirical estimate of population size for any Galapagos shark population. Our recapture rate of 5.4% is consistent with those of other mark-recapture studies. For example, more than half of the shark tagging studies (N = 52) reviewed by [44] reported recapture rates of <5%. A major assumption of closed population models is that the population remains constant over the course of the study. An ongoing multi-species acoustic monitoring study of shark movements throughout the NWHI suggests long-term intra-atoll residency and mixing of Galapagos sharks ([45], C. Meyer unpublished data) and one of eleven Galapagos sharks tagged in 2006 was recaptured during this study (but not included in population models), providing additional evidence of site-fidelity to individual atolls. Our sampling effort was conducted over a short time span (87 days) during summer, minimizing immigration and emigration due to potential seasonal migrations (e.g. [46]). Additionally, Galapagos sharks are long lived species (∼24 yrs, [47]) and neonates were not captured during this study, minimizing the effects of births and deaths. We therefore consider closed population models appropriate for this study, with individual variability models (M_h_) the best fit according to selection criteria. Based on the small number of recaptures, the Chao M_h_ model rather than the Jackknife M_h_ model should provide the most reliable estimates of population size [48]. Due to the small sample size of tagged sharks and relatively wide range of population estimates produced by individual models, our Galapagos shark population estimates should be interpreted cautiously. However, all models were consistent in the magnitude of population size (i.e. hundreds to low thousands).

Population size and CPUE data collectively suggest a relatively small number of large Galapagos sharks use shallow lagoon habitats during the summer months. However, even relatively small numbers of sharks may be a significant source of predation, and therefore natural selection, on species utilizing small sandy islets in these habitats. Many of these species are threatened (Laysan albatross, *Phoebastria immutabilis*), endangered (green turtles, *Chelonia mydas*; blackfoot albatross, *Phoebastria nigripes*) or critically endangered (Hawaiian monk seals, *Monachus schauinslandi*). Within the context of a Marine National Monument, managers must balance conflicting ideological goals of preserving a natural ecosystem, of which predation and natural selection are integral components, with directed management of endangered species. This dichotomy is highlighted in the ongoing debate concerning the culling of sharks to reduce predation on critically endangered Hawaiian monk seals. Low shark abundance in shallow lagoon habitats suggests removal of a small number of sharks from the immediate vicinity of lagoonal islets might significantly reduce short-term predation on monk seal pups without significantly impacting the Galapagos shark population at FFS. However, our results demonstrate very high fishing effort would be required to catch even a few sharks in these areas and we cannot yet predict the duration of lowered predation (i.e. how soon other sharks will move into these habitats). For example, experimental culling of predatory gulls suggested yearly culls would be required to effectively decrease predation rates on other seabirds [49]. In addition, the unintended ecological consequences of shark removal are difficult to predict. Additional empirical data quantifying long-term movements and habitat use of sharks at FFS are needed to assess the likely efficacy and broader ecological impact of culling sharks to reduce predation on monk seals in shallow habitats.
